# Development and assessment of the performance of a shared ventilatory system that uses clinically available components to individualize tidal volumes

**DOI:** 10.1186/s12871-023-02200-2

**Published:** 2023-07-15

**Authors:** David M. Hannon, Tim Jones, Jack Conolly, Conor Judge, Talha Iqbal, Atif Shahzad, Michael Madden, Frank Kirrane, Peter Conneely, Brian H. Harte, Martin O’Halloran, John G. Laffey

**Affiliations:** 1 Department of Anaesthesia, School of Medicine, Galway University Hospitals, University of Galway, Galway, Ireland; 2 Translational Medical Device Lab, University of Galway, Galway, Ireland; 3 Smart Sensors Lab, School of Medicine, University of Galway, Galway, Ireland; 4 School of Computer Science, National University of Ireland Galway, Galway, Ireland; 5 Department of Medical Physics and Clinical Engineering, Galway University Hospitals, Galway, Ireland; 6 CÚRAM Centre for Research in Medical Devices, Biomedical Sciences Building, University of Galway, Galway, Ireland; 7 School of Medicine, Clinical Sciences Institute, University of Galway, Galway, Ireland

## Abstract

**Objectives:**

To develop and assess a system for shared ventilation using clinically available components to individualize tidal volumes.

**Design:**

Evaluation and in vitro validation study

**Setting:**

Ventilator shortage during the SARS-CoV-2 pandemic.

**Participants:**

The team consisted of physicians, bioengineers, computer programmers, and medical technology professionals.

**Methods:**

Using clinically available components, a system of ventilation consisting of two ventilatory limbs was assembled and connected to a ventilator. Monitors for each limb were developed using open-source software. Firstly, the effect of altering ventilator settings on tidal volumes delivered to each limb was determined. Secondly, the impact of altering the compliance and resistance of one limb on the tidal volumes delivered to both limbs was analysed. Experiments were repeated three times to determine system variability.

**Results:**

The system permitted accurate and reproducible titration of tidal volumes to each limb over a range of ventilator settings and simulated lung conditions. Alteration of ventilator inspiratory pressures, of respiratory rates, and I:E ratio resulted in very similar tidal volumes delivered to each limb. Alteration of compliance and resistance in one limb resulted in reproducible alterations in tidal volume to that test lung, with little change to tidal volumes in the other lung. All tidal volumes delivered were reproducible.

**Conclusions:**

We demonstrate the reliability of a shared ventilation system assembled using commonly available clinical components that allows titration of individual tidal volumes. This system may be useful as a strategy of last resort for Covid-19, or other mass casualty situations, where the need for ventilators exceeds supply.

**Supplementary Information:**

The online version contains supplementary material available at 10.1186/s12871-023-02200-2.

## Article Summary

### Strengths and limitations of this study

- Our solution demonstrates the potential to ventilate two patients simultaneously while delivering differing tidal volumes in each circuit, using equipment readily available in most hospitals.
- Accurate and reproducible titration of tidal volumes to each ‘lung’ was possible over a wide range of ventilator settings, and under conditions of varying compliance and resistance.
- Alteration of one simulated ‘lung’ conditions had minimal impact on the tidal volumes delivered to the unaffected lung.
- The system relies on patients being sedated and paralysed.
- The system does allow for measurement of peak and plateau pressures in individual patients, though a complex intervention to isolate each patient is required.

## Introduction

The global pandemic of coronavirus disease caused by the SARS-CoV-2 virus began in late 2019. The pandemic placed unprecedented pressures on Intensive Care Units (ICUs) worldwide. Many cases were complicated by acute severe pulmonary failure [1], and as many as 20% of hospitalized patients required ICU admission [2, 3]. Approximately half of these were likely to require mechanical ventilation in the earliest phases of the pandemic. The resulting pressure led to difficult ethical decisions regarding resource allocation [4]. One solution to this issue is to ventilate more than one patient from a single ventilatory source.

Most patients with Covid-19 admitted to an ICU fulfil the criteria for a diagnosis of Acute Respiratory Distress Syndrome (ARDS) [5]. A key recommendations for the optimal respiratory management of mechanically ventilated patients with ARDS is the implementation of a strategy of ‘lung protective’ ventilation [6]. This involves mechanical ventilation that effectively oxygenates the patient while avoiding further injury to the lungs through high pressures or volumes that will lead to inflammatory damage [7], otherwise known as Ventilator Induced Lung Injury (VILI). The key components of effective lung protective ventilation include the delivery of tidal breaths of approximately 6ml/kg of predicted body weight [8], and the maintenance of a plateau airway pressure below 30cmH_2_O [9, 10]. In the context of shared ventilation, this necessitates a system in which each circuit can deliver individualised and titratable tidal volumes for the patient being ventilated, in the settings of alterations in lung compliance and resistance.

Prior to the pandemic, ventilation of two ‘patients’ with a single ventilator has been the subject of equipment [11, 12] and animals tests [13], computer simulation [14, 15], and has been utilised in a clinical setting [16]. A limitation to these systems is the inability to provide individualised tidal volumes to each patient. In these referenced approaches, the ventilator delivers a single breath which is then ‘equally’ divided among the patients attached to the circuits. Some variations will take place because of the individual lung mechanics of these patients, but the systems lack the ability to intentionally titrate the resulting tidal volumes in each part of the circuit. The result is that patients must be closely matched and monitored along a range of physiological variables for lung-protective ventilation to be employed correctly. Best practice in mechanical ventilation for patients with ARDS requires the ability to adjust tidal volumes based on patient characteristics [10], and a system where different volumes could be delivered in separate circuits from the same source would be ideal.

Ventilator sharing is a strategy of last resort, though reports of doctors obliged to choose which patients should receive ventilation underline the need to consider this strategy as part of a response that increases short term ventilator capacity. Our objective in this study was to develop a simple, reliable system that permits shared ventilation using commonly available clinical components, and that allows titration of tidal volumes under conditions of alterations in lung compliance and resistance. This study evaluates the performance of this shared system throughout a range of clinically relevant parameters.

## Methods

### Setting

The system was tested using an ICU ventilator attached to the ventilation system. Each circuit ventilated a test lung. The investigations were performed in a laboratory setting using test equipment.

### Components and Assembly

**Fig. 1:**
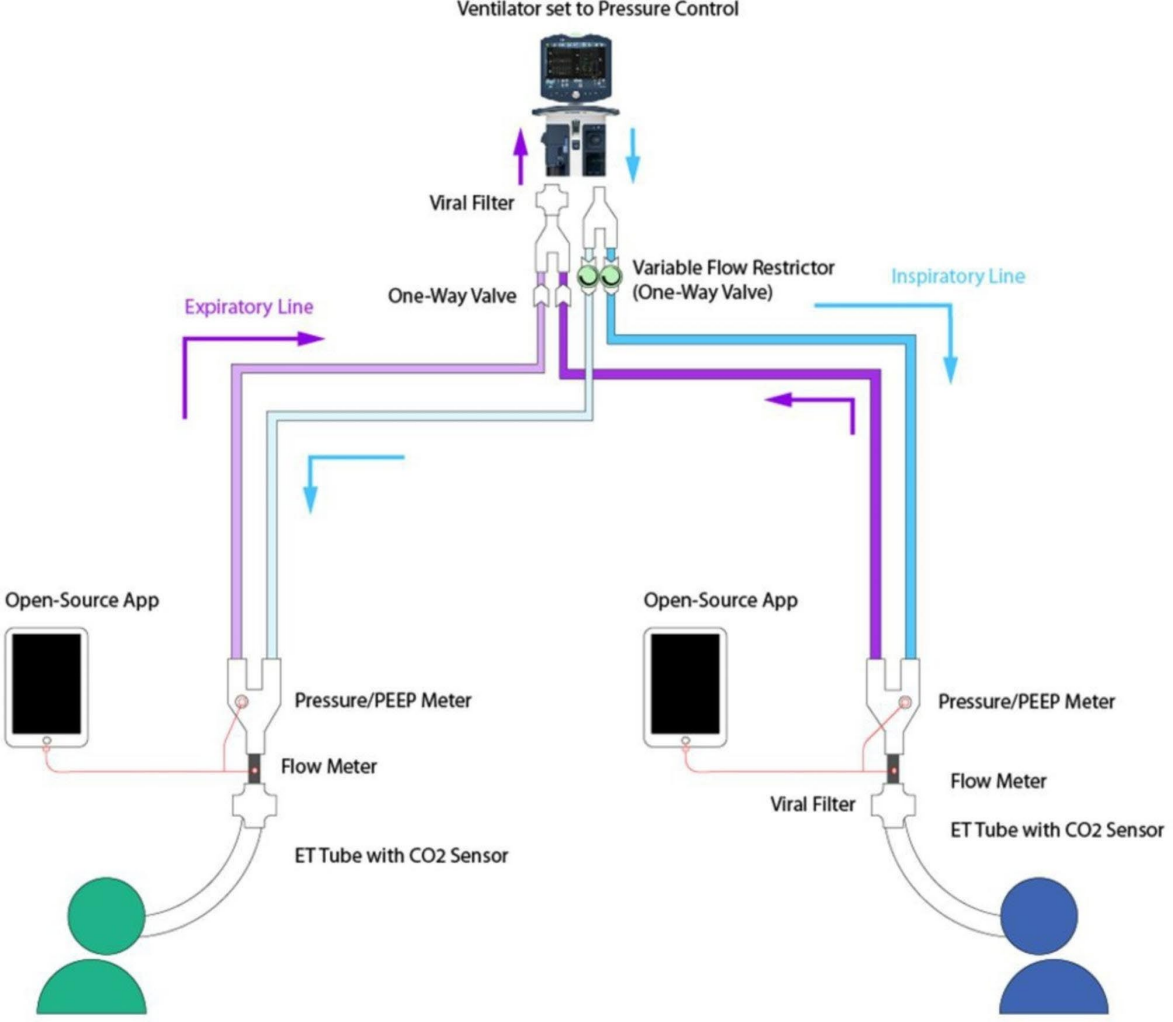
Diagram of the assembled shared ventilation system. Further details of the exact components used, and their assembly, are available in the supplementary material

**Fig. 2:**
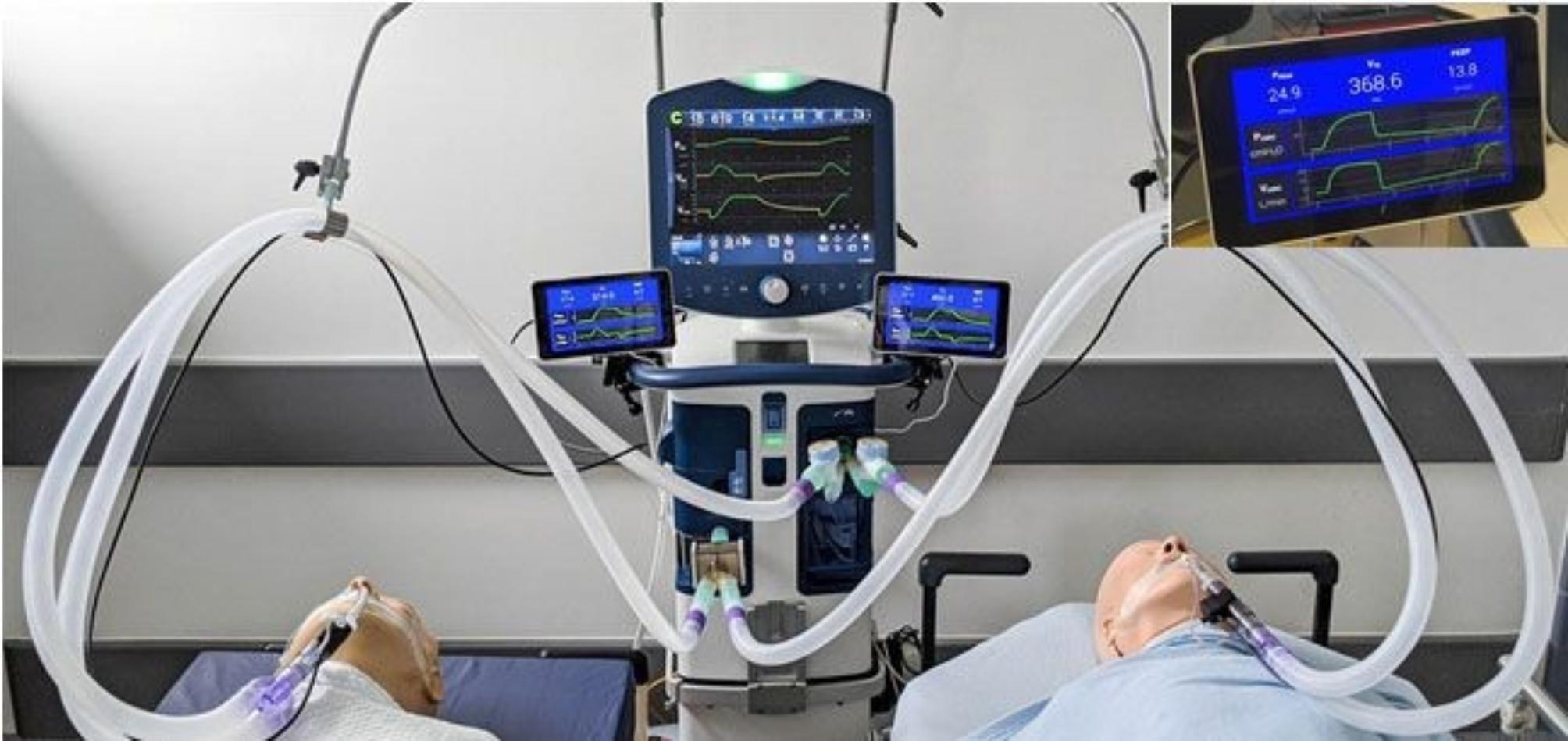
VentShare System attached to two mannikins. Monitoring of the respiratory parameters for each limb are shown to the right and left of the main ventilator. A closeup of the display readout is also shown

The system was assembled as per the schematic design in Fig. 1. A full list of components and assembly instructions are available in Appendix A. The ventilator used was a Puritan Bennet 980 (Medtronic plc). The ventilator operated in Pressure Control mode throughout the tests. Each limb was connected to a Training and Test Lung (Michigan Instruments Inc). Tidal volumes and pressures was captured with the Citrex H4 gas flow analyser (IMT Analytics AG) and a custom developed open-source solution developed using a pressure sensor processed through the Python programming language. The monitoring system consists of a gas flow sensor (Sensirion, SFM3200/3300), a pressure sensor (Analog Microelectronics, AMS-5915-0200-D-B), USB sensor cable (Nicolay GmbH), a processing and display unit (Raspberry Pi, 7-inch standard screen), and software (open-source creative commons license). An image of the assembled system is shown in Fig. 2.

**Fig. 3:**
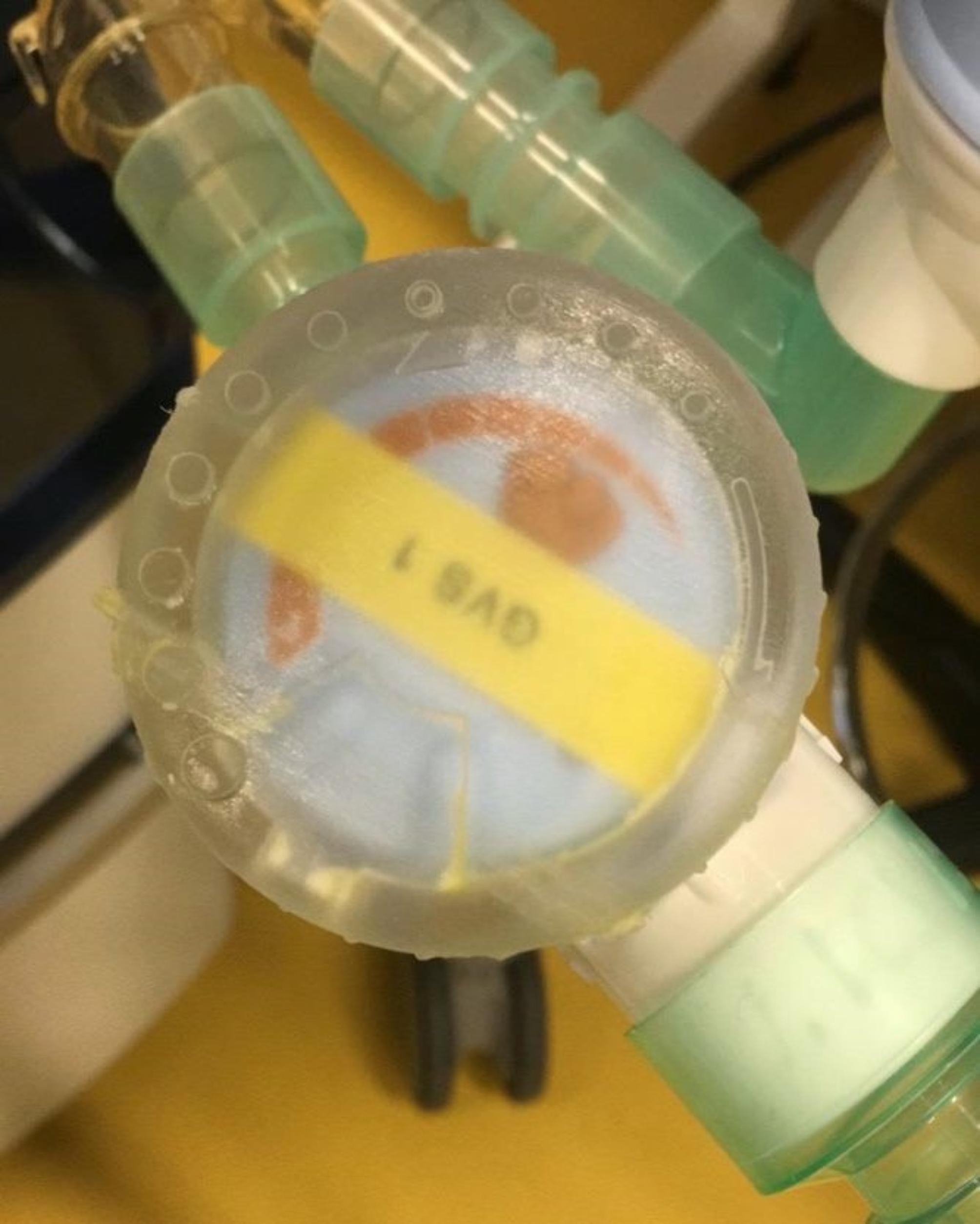
Photograph of control cap custom designed and applied to the top of the APL valves to allow more precise control and testing of the shared circuit

To assist with accuracy in testing the positions of the Adjustable Pressure-Limiting (APL) valves, plastic covers were fitted (Fig. 3). These covers were developed by the firm Design Partners (Dublin, Ireland). These covers were marked with ten equidistant dots to allow greater precision and repeatability in positioning the APL valves. Position 0 corresponded to fully open, and position 10 to fully closed.

### Procedure

The system was subjected to a series of repeated tests. These tests fell into two groups. In the first group of tests (Test 1a, 1b, 1c), the impact of altering the settings of the ventilator on the tidal volumes delivered to each test lung was determined. In the second group (Test 2a, 2b), the system performance under different conditions of compliance and resistance in each limb of the circuit was recorded. Each of these tests was repeated three times. The limbs of the circuit will henceforth be referred to as ‘Limb A’ and ‘Limb B’.

With each variation of settings, the following parameters were recorded:

- **ventilator**
  - Inspiratory Pressure (cmH_2_O).
  - Positive End Expiratory Pressure (cmH_2_O).
  - Respiratory rate (breaths per minute).
  - Inspiratory: Expiratory ratio.
- **circuits (A & B)**
  - Compliance of test lung (ml/cmH2O).
  - APL closure setting (0–10).
  - Tidal volume (mL).
  - Peak pressure (cmH_2_O).
  - Positive End Expiratory Pressure (cmH_2_O).

### Alteration of Ventilator Settings (Test 1a, 1b, 1c)

The compliance of the test lung for Circuit A and Circuit B was 10 ml/cmH_2_O. The APL valve on each circuit was set to mark 3 of 10. Standard ventilator settings were a respiratory rate (RR) of 14 breaths per minute, positive end expiratory pressure (PEEP) of 8 cmH_2_O, inspiratory pressure for each breath was 10cmH_2_O, and the I:E ratio was 1:2.

### Test 1a – Alteration of inspiratory pressure

In these tests, the inspiratory pressure delivered by the ventilator was altered. Initial inspiratory pressure was 5 cm H_2_O. This was raised by 1 cm H2O and observations recorded. Observations were recorded for inspiratory pressures between 5 and 20cmH_2_O.

### Test 1b – Alteration of respiratory rate

In this group, the respiratory rate of the ventilator was initially set to 6 breaths per minute. The respiratory rate was increased by 1 and observations recorded. The respiratory rate was altered between 6 and 20 breaths per minute.

### Test 1c – Alteration of I:E ratio

In these experiments, the inspiratory to expiratory ratio was altered. The initial ratio was 1:1. Observations were recorded, and the ratio was increased by 0.5 (creating a series of ratios of 1:1, 1:1.5, 1:2, etc.). This was continued to an I:E ratio of 1:4.

### Alteration of each circuit condition (Test 2a, 2b)

In the second group of experiments, in which effects to changes of Circuit A were recorded on the system, the ventilator settings were: inspiratory pressure of 10 cmH_2_O, RR was 14 breaths per minute, PEEP was 5 cmH_2_O, and I:E ratio was 1:2. Unless otherwise altered, compliance for each circuit was 10 ml/cmH_2_O.

### Test 2a – Alteration of test lung compliance

In this group, the compliance of Circuit A was altered from 10 ml/cmH_2_O to 150 ml/cmH_2_O. At each step the compliance was increased by 10 ml/cmH_2_O. The APL valve of Circuit A and Circuit B was set to 3 of 10.

### Test 2b – Alteration of APL closure

In this batch, lung compliance for each circuit was set to 10 ml/cmH_2_O. The APL of Circuit B was fully open, and the APL of Circuit A was manipulated in a series of steps from 0 (fully open) to 10 (fully closed), increasing by 1 at each step.

### Statistical analysis

Statistical analysis was performed using Sigmaplot 10.0 (Systat Software, San Jose, CA, USA). The measured variable for all interventions was tidal volume. Data were analyzed using two-way analysis of variance (ANOVA), with ventilation circuit, and the experimental intervention (alterations in inspiratory pressure, respiratory rate, I:E ratio, circuit compliance, resistance) as factors with post hoc comparisons using Student-Newman-Keuls. The ⍺ level for all analyses was set as p < 0.05.

Data processing and graphs were generated using the open-source statistical programming package R, version 4.0.2.

### Patient and public involvement

Patients were not involved in the development or testing of this evaluation of a configuration of equipment.

## Results

### Alteration of ventilator settings

#### Effect of alterations in Inspiratory Pressure

**Fig. 4:**
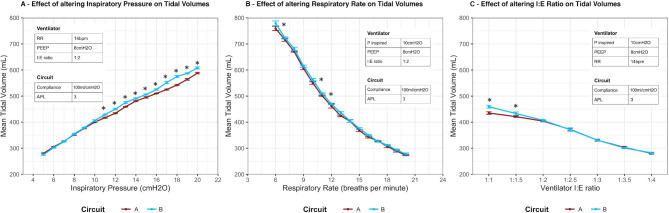
Effect of altering ventilatory parameters on tidal volumes delivered to simulated lungs ‘A’ and lung ‘B’

The increase in inspiratory pressure produced a progressive increase in tidal volume in both circuits (Fig. 4 ***Panel A***). The variability within each circuit across the three repetitions was low, as evidenced by the low standard deviations for each measured tidal volume at each inspiratory pressure (Fig. 4 ***Panel A***). The variability in tidal volumes across the range of inspiratory pressures varied from under 1% to a maximum of 6% of tidal volume. At several inspiratory pressures, these differences were of statistical significance (p < 0.05).

#### Effect of alterations in respiratory rate

The stepwise increase in respiratory rate (RR) resulted in a progressive decrease in tidal volume in both ventilator circuits (Fig. 4 ***Panel B***). The variability within each circuit was low, as evidenced by the low standard deviations for each measured tidal volume at each rate setting in both circuits (Fig. 4 ***Panel B***). The variability in tidal volumes across the two circuit circuits across the range of inspiratory pressures ranged from < 1% to a maximum of 3% of delivered tidal volume. These differences reached statistical significance (p < 0.05) at multiple RRs.

#### Effect of alteration of I:E ratio

The stepwise decrease in I:E ratio resulted in a progressive decrease of tidal volume in both ventilator circuits (Fig. 4 ***Panel C***). The variability within each circuit was low, as evidenced by the low standard deviations for each measured tidal volume at each I:E setting in both circuits (Fig. 4 ***Panel C***). The variability in tidal volumes across the range of inspiratory pressures ranged from < 1% to a maximum of 5% of delivered tidal volume. Again, these differences reached statistically significance (p < 0.05) at higher I:E ratios.

### Alteration of individual circuit settings

#### Alteration of test lung compliance

**Fig. 5:**
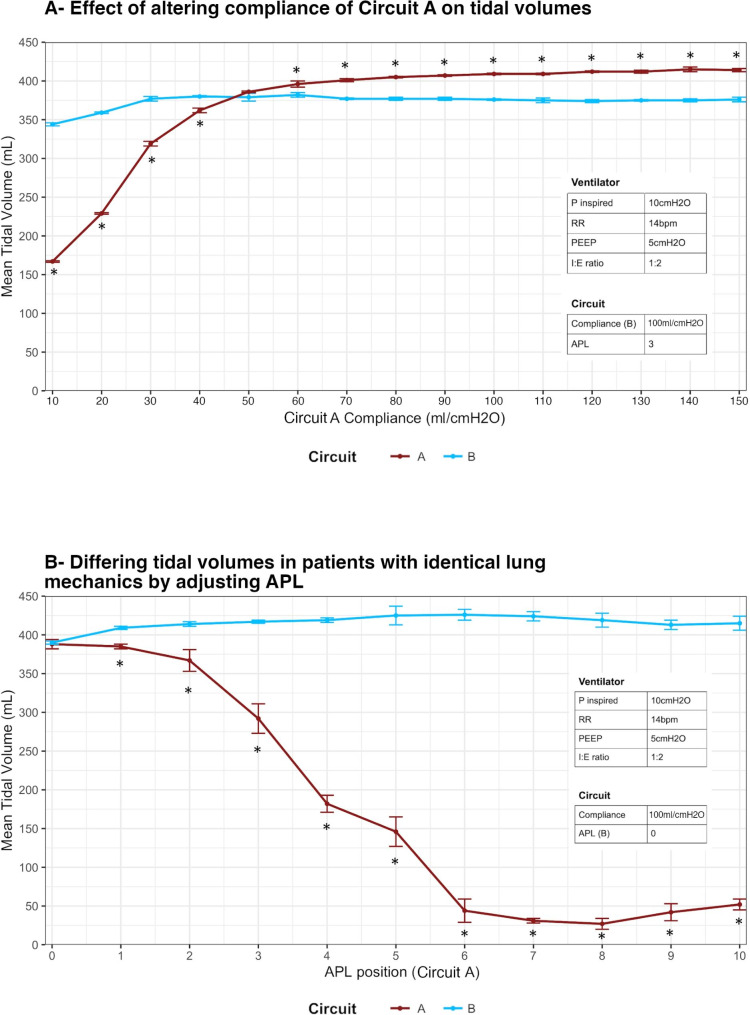
Effect of altering the conditions of one simulated ‘lung’ (Lung A) on tidal volumes delivered to both Lung A and Lung B

Progressively increasing the compliance of the ‘lung’ in Circuit A, while keeping the compliance in ‘lung B’ constant, led to a significant increase in tidal volume delivered to Circuit A for each increment in lung compliance (Fig. 5 ***Panel A***). In contrast, the effect on tidal volume delivered to Circuit B was limited. There was a significant increase in tidal volume delivered to the circuit with initial increments in the compliance of lung A, but thereafter there was no significant change in tidal volume delivered to lung B (Fig. 5 ***Panel A***). The variability with each circuit at each level of compliance of lung A was low, as evidenced by the low standard deviations for each measured tidal volume at each compliance setting.

#### Alteration of circuit Resistance via APL closure

Progressively increasing the resistance of the circuit in Circuit A (by closing the APL valve), while keeping the resistance of the circuit in Circuit B constant, led to a significant decrease in tidal volume delivered to Circuit A for each incremental change in circuit resistance (Fig. 5 ***Panel B***). In contrast, the effect on tidal volume delivered to Circuit B was limited. There was a significant decrease in tidal volume delivered to Circuit A with increases in the resistance of Circuit A, but thereafter there was no significant change in tidal volume delivered to Circuit B (Fig. 5 ***Panel B***). The variability with each circuit at each level of resistance of the circuit in Circuit A was low, as evidenced by the low standard deviations for each measured tidal volume at each resistance setting.

## Discussion

Our experimental findings demonstrate that we have constructed a system that enables shared ventilation and allows titration of tidal volumes independently in each circuit using a paediatric APL valve to create variable resistance to flow. Our findings demonstrate that changes in resistance and compliance in one circuit have minimal effects on the other. The system allows tidal volumes that are titratable and are stable for long periods, having been stable for over 24 h at various settings. Given the heterogenous nature of lung mechanics involved in Covid-19 patients [17], the ability to titrate tidal volumes is key in ensuring that mechanical ventilation will not further damage already compromised lungs [18].

In practice, the APL on the circuit in which the test lungs were *least* compliant was left open to create minimal resistance to flow. The desired tidal volume was created in this circuit by manipulating the inspiratory pressure on the ventilator directly, and then closing the APL valve on the other circuit to generate resistance to flow that would result in the desired tidal volume in that circuit being attained. Future tests are set to evaluate the system in scenarios that closely resemble actual clinical use cases, and the main objective of this work was to outline the performance envelope of the system. It is possible to examine the recorded tests and draw conclusions about the clinical potential of the system. During our investigations we had no difficulty in achieving identical tidal volumes in two different circuits – one with compliance of 50ml/cmH2O and the other with compliance 20ml/cmH2O by closing the APL valve in the circuit with the greater compliance whilst keeping the other in the fully open position (see Table A02 in Online Supplement). The effects of these changes can be seen where the difference in tidal volumes between two compliances, with both APLs fully open, can be seen. The target tidal volume, in the limb with the lowest compliance, is then achieved by manipulating the inspiratory pressure. Finally, the APL valve of the circuit with the higher compliance is manipulated to achieve a resistance allowing that circuit to achieve a relevant tidal volume. In this way, the system allows for identical or different tidal volumes to be delivered to lungs with different compliances.

As outlined in the results, statistical significance was reached at multiple points when analysing the difference in tidal volume between Circuit A and B, under circumstances where there would ideally be none. Whilst statistical significance was reached, these differences would be of limited clinical relevance, and reflects small differences in circuit characteristics, combined with low inter-test variability.

The testing outlined in this paper used a critical care ventilator, but the system has also been tested on a range of ventilators and can be utilised with sources that are capable of Pressure Control ventilation. The use of Volume Control ventilation can result is unpredictable delivery of tidal volumes to each circuit and therefore risks hyperinflation of areas of lung [19]. An important aspect of lung-protective ventilation relates to limiting the pressures that the ventilated lung is subjected to [10]. Although the use of the system in pressure control mode does allow one to place limits on the maximum pressure delivered to the lung, it is important to be able to measure the plateau and driving pressures in each circuit [20]. This requires utilising inspiratory and expiratory hold manoeuvres. The use of these has not been fully investigated and documented with this system. Executing such an action would require multiple people coordinating to simultaneously isolate one patient by clamping their ETT whilst simultaneously closing the APL valve of that circuit, whilst performing the hold manoeuvre on the other. The logistics and coordination required for this, without even considering the safety, are likely to be challenging in a context which, by definition, is operating at or beyond normal limits of capacity. Given that the correct use of lung-protective ventilation reduces mortality in ARDS [8], this forms on important area of further investigation. Further practical simulation studies investigating the practical limitations of the system are, indeed, ongoing. This will include delivering similar tidal volumes through a range of altered I:E ratios and respiratory rates.

Any system of shared ventilation allows for the ‘expansion’ of equipment stocks and enables life-preserving therapy for multiple patients. However, these systems also have significant limitations. These drawbacks have even led medical organisations to advocate against the use of shared ventilation [21]. However, we feel that further exploration of this area is an important step in a ‘last resort’ scenario.

It important to note that the technical drawbacks of this system, and the equipment it necessitates, are often not the limiting factor in delivering ventilatory support to critically ill patients in resource-poor settings [22], where the ability to delivery supplemental oxygen is often the most important constraint [23]. The most effective strategies in these settings often involve halting the spread and severity of the disease before it leads to critical illness [24]. As such, shared ventilation may be better suited to environments with relevant expertise, but limited equipment stocks. The monitoring system used is a custom solution developed using commonly available parts from multiple online and regional local resellers worldwide. There are other systems available that seek to use the information obtained from an arterial pressure transducer to achieve these aims [25] and this could enable an alternative solution to this issue than the one we present here.

Any discussion of shared ventilation must also acknowledge the ethical implications inherent in this strategy. The approach necessitates depriving a single patient of ‘standard-of-care’ treatment to attempt the preservation of two lives [26]. Taking this action in the absence of robust data regarding the efficacy of shared ventilation for Covid-19 patients is challenging, as one cannot be certain that it will confer benefit. Despite the attempts that have been made to aid these decisions [27], the initiation of shared ventilation is an ethically challenging act.

Despite the progress our solution represents, there are still multiple limitations that must be considered when evaluating potential clinical use. Employing this arrangement demands a high level of training, and usually comes with significant restrictions regarding the ability to alter respiratory parameters for each patient [19]. High levels of skill and knowledge are needed to operate any system involving shared ventilation [28], emphasising the importance of training and coordination between multiple team members in order to fully and safely operate a system such as this. The authors are engaged in investigating and developing this. The authors do not advocate ventilator sharing as a normal course of therapy. Our aim is to provide details of a system that can be used as a strategy of last resort by medical professionals who find themselves in a situation in which only shared ventilation can preserve life. In addition, patients must still share most ventilation parameters. This includes identical respiratory rates, positive end expiratory pressures, I:E ratio, and fraction of inspired oxygen. The authors are continuing to explore solutions to these issues, as they are factors continuing to restrict the scope of patients that can be initiated on shared ventilation [29]. Given the shared nature of the circuit, both patients must be fully paralysed, as there is no facility to allow spontaneous in this configuration. Paralysis is not helpful beyond short time-frames [30] in a critical care patient, and the risk of undue muscular atrophy must be carefully considered when embarking on a course of care that necessitates it. An ideal solution would allow the titration of as many respiratory parameters as possible, could respond to changes in lung mechanics of patients, whilst facilitating lung protective ventilation. Although the system remains stable over the most APL settings, a relative decrease in PEEP can be seen when the APL moves beyond point 5. This limits the utility of the system as might be the case if the two patients who are sharing the system have very disparate tidal volume targets, or if there were significant changes in lung compliance. The loss of PEEP at these extremes can have important implications for patients with Covid-19, as the lungs of these patients are often highly responsive to PEEP [31]. If the two patients have been matched based on similar PEEP requirements, a large difference in this value could be detrimental. In addition, when both APL valves were closed beyond position 5 simultaneously, a degree of resistance to flow was generated that usually resulted in pressure alarms in the ventilator being activated. While this could have been overcome by overriding certain settings with the assistance of the equipment manufacturer, this was deemed unnecessary as these high resistance scenarios were of limited relevance to COVID ARDS.

A final important potential limitation is the possibility of cross-contamination of pathogens between the circuits. Multiple attempts to mitigate this possibility are present in the system. Between the tubing of the inspiratory limbs and the endotracheal tubes, heat and moisture exchange filters are utilised. These have been shown to be effective in preventing SARS-CoV-2 spread in mechanically ventilated patients with Covid-19 [32]. The expiratory limbs unite to return to the ventilator. Gas flows through a high-efficiency particulate air (HEPA) filter before returning to the ventilator, ensuring that any viral particles are not allowed to return to the machine [33]. To ensure no mixing of air between the circuits takes place when the inspiratory and expiratory limbs reunite, one-way flow valves are employed. Despite our confidence that these measures mitigate against cross-contamination of gas between the circuits, it must be noted that this has not been formally assessed, and we cannot be certain that cross-contamination is impossible without further testing.

## Conclusions

We have shown that a system of shared ventilation using commonly available components that allows titration of tidal volumes is possible and easily assembled. The detailed monitoring of ventilatory support delivered to each patient remains important and is now possible using cheap equipment and open-source software.

Further work is necessary to determine ways in which additional respiratory parameters can be monitored and altered in each of the circuits. In conclusion, we must maintain the position that multiple patients should not be placed on a single ventilator in anything other than a crisis.

In circumstances that require the preservation of life, we believe our solution offers an important innovation toward expanding this strategy. However, important areas remain to be explored and resolved before a system such as this could be deployed in a clinical environment with real patients.

## Electronic supplementary material

Below is the link to the electronic supplementary material.

Figure A01. Stepwise assembly of ventilation system. Table A01. Equipment list for the full assembly of the Galway VentShare system. Table A02: Variation of tidal volume as function of altering compliance and resistance across both circuits

## Data Availability

Test data can be accessed as a supplement to this article, or by reasonable request to David M Hannon (d.hannon8@universityofgalway.ie).

## Declarations

**Ethics statement**.
