# Supplementary material for: Development and assessment of the performance of a shared ventilatory system that uses clinically available components to individualize tidal volumes
Source: BMC Anesthesiol. 2023 Jul 15;23:239. doi: 10.1186/s12871-023-02200-2 (PMC10349497; doi:10.1186/s12871-023-02200-2)
Supplement: Supplementary file 1 — Figure A01. Stepwise assembly of ventilation system. Table A01. Equipment list for the full assembly of the Galway VentShare system. Table A02: Variation of tidal volume as function of altering compliance and resistance across both circuits [file 12871_2023_2200_MOESM1_ESM.docx]

# ONLINE SUPPLEMENT

## Title Development and assessment of the performance of a shared ventilatory system that uses clinically available components to individualize tidal volumes

## Authors: David M. Hannon^1,2^, Tim Jones^2^, Jack Conolly^2^, Conor Judge^2^, Talha Iqbal^3^, Atif Shahzad^3^, Michael Madden^4^, Frank Kirrane^5^, Peter Conneely^5^, Brian H. Harte^1^, Martin O’Halloran^2,6^, John G. Laffey^1,2,6^

#
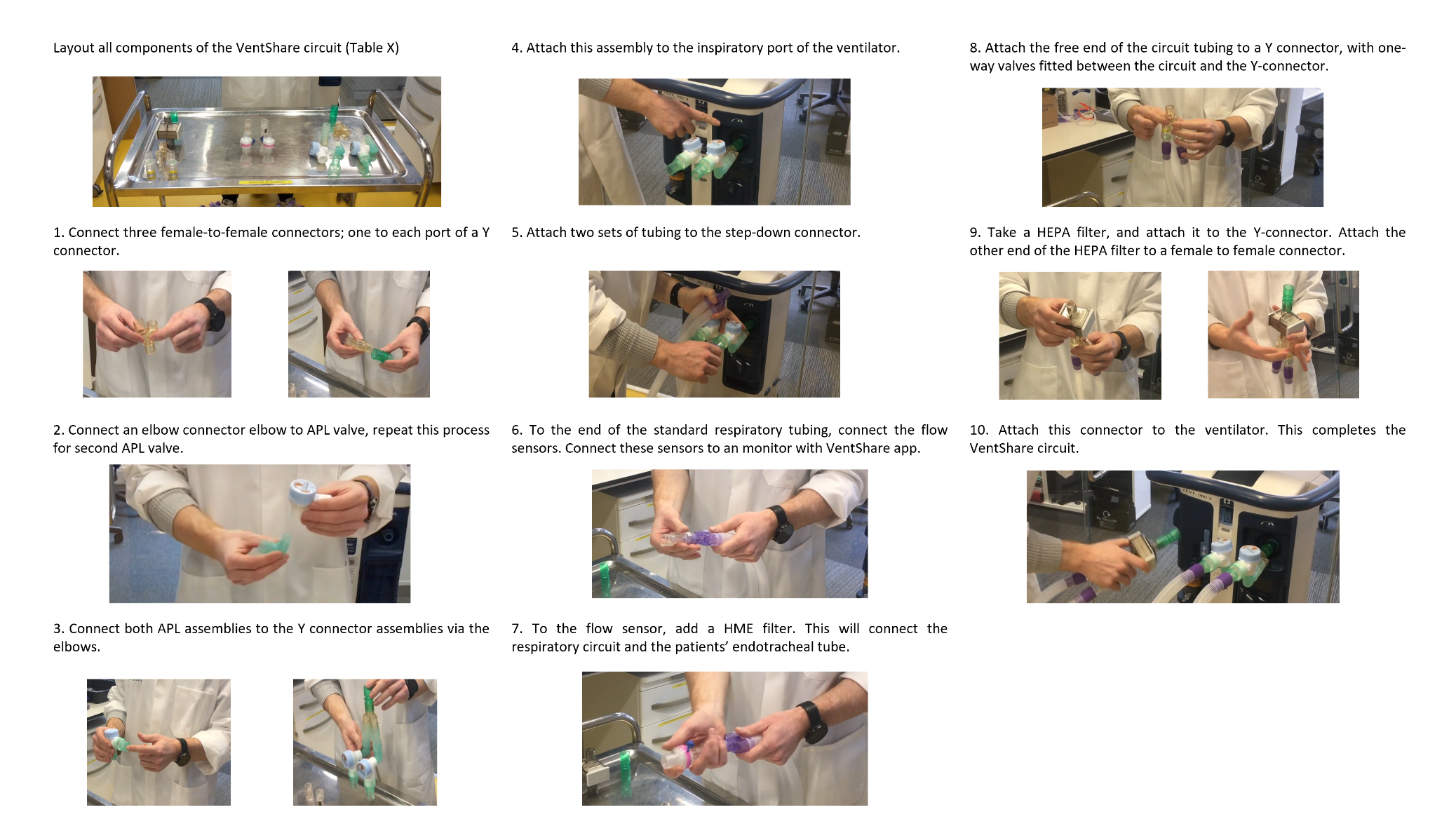
Appendix A – System Assembly

**Figure A01. Stepwise assembly of ventilation system**

| **Quantity** | **Item** | **Description** |
| --- | --- | --- |
| 1 | Ventilator | An ICU ventilator offers the greatest control and sophistication, but the VentShare system has been tested on simpler ventilators and has shown comparable performance. |
| 2 | Ventilator circuit | Ensuring that these circuits are of sufficient length to reach each patient, but as short as possible within this constraint in order to minimise apparatus dead space. |
| 2 | Y-connector | 22mm male to 2 x 22mm male connectors |
| 2 | APL Valve | Paediatric APL valves from Mapleson anaesthetic circuits have been found to give reliable performance in testing. Importantly, these valves must incorporate a 30mm scavenging port. |
| 2 | Elbow connector | Connect directly to the APL valves and to the respiratory tubing. We have used 15mm male to 22mm male connectors. |
| 2 | Step-down connector | 30mm female to 22mm male connectors. |
| 4 | Female-to-female connector | Allows for connection of 22mm male to 22mm male connectors. |
| 2 | Flow sensors | The sensors used are manufactured by Sensirion. They are not necessary for operation of the circuit but are recommended to ensure accurate monitoring of each limb. |
| 2 | Heat and moisture exchange filter (HMEF) | We recommend HMEs that are capable of removing both bacteria and viruses (i.e. a dry circuit). |
| 1 | High Efficiency Particulate Air filter  (HEPA filter) | This type of air filter can theoretically remove greater than 99.97% of any airborne particles with a size of 0.3 microns (µm). |
| 2 | One-way valves | Mounted on the expiratory limb, these are key to minimise ‘cross-talk’ between the individual limbs of the circuit. |
| 2 | Control Cap | Plastic caps covering the APL dials, providing more detailed visual feedback for the degree of APL closure. |

**Table A01. Equipment list for the full assembly of the Galway VentShare system.**

| **Time Point/Step** | | **1** | **2** | **3** | **4** | **5** |
| --- | --- | --- | --- | --- | --- | --- |
| **Vent. Inspiratory Pressure (cmH2O)** | | 15 | 15 | 15 | 19 | 19 |
| **Circuit A** | **Set Compliance**  **(mL/cmH2O)** | 50 | 20 | 20 | 20 | 20 |
|  | **Set Resistance (APL setting)** | 0 | 0 | 0 | 0 | 0 |
|  | **Delivered Vt (mL)** | 442 | 293 | 295 | 458 | **449** |
|  |  |  |  |  |  |  |
| **Circuit B** | **Set Compliance**  **(mL/cmH2O)** | 50 | 50 | 50 | 50 | 50 |
|  | **Set Resistance (APL setting)** | 0 | 0 | 4 | 4 | 3 |
|  | **Delivered Vt (mL)** | 464 | 468 | 302 | 352 | **439** |

**** NB ventilator settings, other than written above, were PEEP 10cmH2O, RR 14 bpm, and I:E 1:3***

**Table A02: Variation of tidal volume as function of altering compliance and resistance across both circuits**
